# Outcome of Bilateral Hand Reconstruction in a Child Presenting Late With Apert Syndrome: A Case Report and Literature Review

**DOI:** 10.7759/cureus.43641

**Published:** 2023-08-17

**Authors:** Brandon Lim, Mohamed Shalan

**Affiliations:** 1 School of Medicine, Trinity College Dublin, Dublin, IRL; 2 Orthopaedics and Traumatology, The Mater Misericordiae University Hospital, Dublin, IRL; 3 Trauma and Orthopaedics, St. James's Hospital, Dublin, IRL

**Keywords:** apert hands, congenital hand defect, syndactyly, hand reconstruction, apert syndrome

## Abstract

Apert syndrome is a rare inherited syndrome characterised by craniosynostosis, midface hypoplasia, and syndactyly of the hands and feet. Syndactyly of the hands is categorised into three types with varying severity, requiring a diverse range of surgical techniques to produce good functional and aesthetic outcomes. The best age to initiate hand reconstruction is between three and 12 months. We present a case of a three-year-old boy with type III syndactyly who first presented at a volunteer outreach surgical campus in Pemba, Zanzibar. A three-stage bilateral hand reconstruction was initiated to sequentially create the first, fourth, and second web spaces. Postoperative healing was uneventful. He underwent a hand rehabilitation program and demonstrated good functional outcomes, being able to attend school, hold a pen, and write by seven years old. A literature review revealed that the best age to initiate hand reconstruction or the best surgical technique to use has yet to be agreed upon. It is agreed that the diverse symptoms of Apert syndrome make it difficult to manage, requiring multidisciplinary collaboration to provide physical and emotional benefits to patients and families.

## Introduction

Apert syndrome is a rare genetically inherited syndrome with characteristics such as craniosynostosis, midface hypoplasia, and syndactyly of the hands and feet, occurring in 1/160,000-200,000 live births [1-6]. It is more common in Asia [1,2], and its incidence increases with paternal age [3,5]. It can be inherited in an autosomal dominant manner or through a sporadic mutation involving a gain-of-function missense mutation of fibroblast growth factor receptor 2 (FGFR2) on chromosome 10q [2,3,5]. Mutations in the binding site between immunoglobulin-like loops 2 and 3 on FGFR2 lead to deficiencies in intracellular signals that regulate embryogenesis, causing premature gastrulation, implantation anomalies, impaired epithelial-mesenchymal interactions, and defective membranous and endochondral bone formation [4].

Upton (1991) classified the syndactyly in Apert syndrome into three types, ascending in rarity and severity: type I - spade hand or obstetrician's hand; type II - mitten hand; and type III - the hoof or rosebud [7]. All three types have thumb-radial clinodactyly, complex 2-3-4 syndactyly, and symbrachyphalangism; types II and III have index-radial clinodactyly but type I does not; type I and II have simple, non-osseous first web syndactyly and simple, incomplete 4-4 syndactyly while type III has complex, osseous first web syndactyly and simple, complete 4-4 syndactyly [3].

The management of syndactyly in Apert syndrome involves hand reconstruction surgery, with the optimum age for surgery being subject to debate, ranging from three to 12 months of age [3,8,9]. Furthermore, surgical techniques to release syndactyly in the management of Apert syndrome are varied because of the variety of presentations and the complex involvement of the soft and hard tissues of the hand [6]. Post-surgical complications include infections, maceration of flaps or grafts, graft failure, web creep, and hypertrophic scars [6].

## Case presentation

We present a case of a three-year-old boy presenting with craniofacial dysmorphism and syndactyly of the hands and feet. This patient was seen and operated on at a volunteer surgical campus in Pemba, Zanzibar. This outreach programme has been carried out biannually since 2014. Many African countries are underdeveloped and financially poor, suffering from inadequate facilities while surgical implants and instruments are unavailable. As such, volunteers needed to carry all the necessary tools with them.

**Figure 1:**
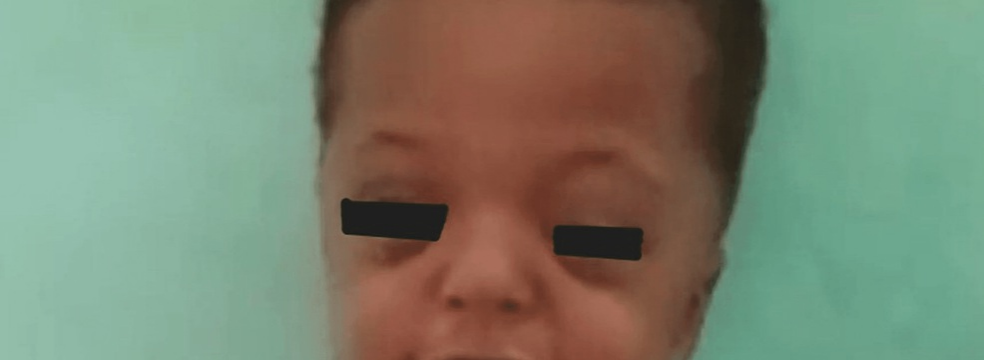
Facial features include a prominent forehead, ocular hypertelorism, downward palpebral slant, flat nasal bridge, and bulbous nose

**Figure 2:**
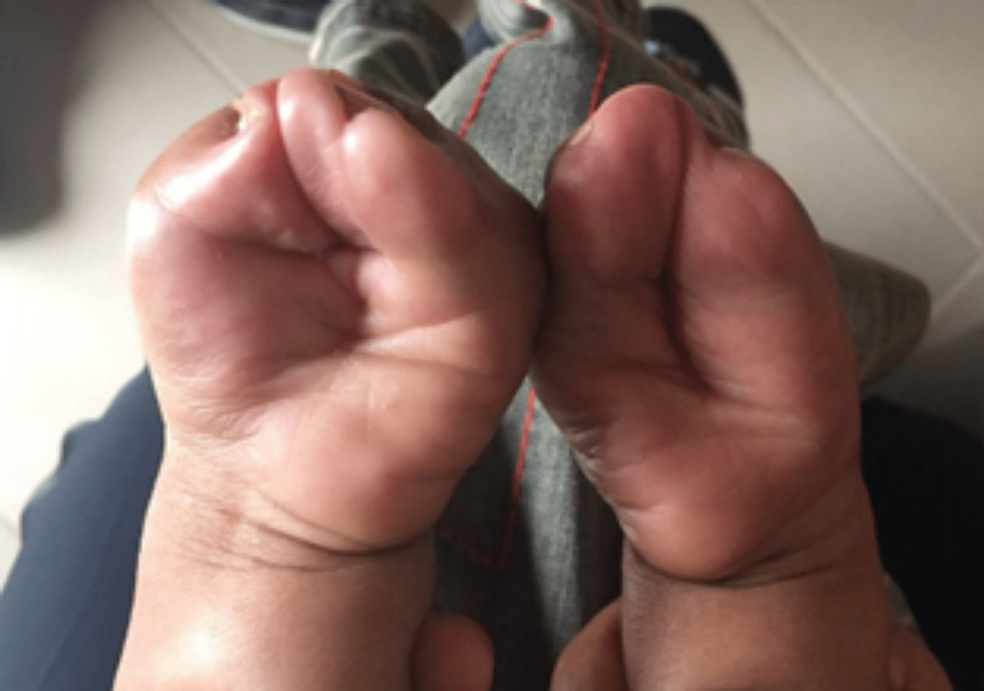
Bilateral symmetrical syndactyly of the hands

**Figure 3:**
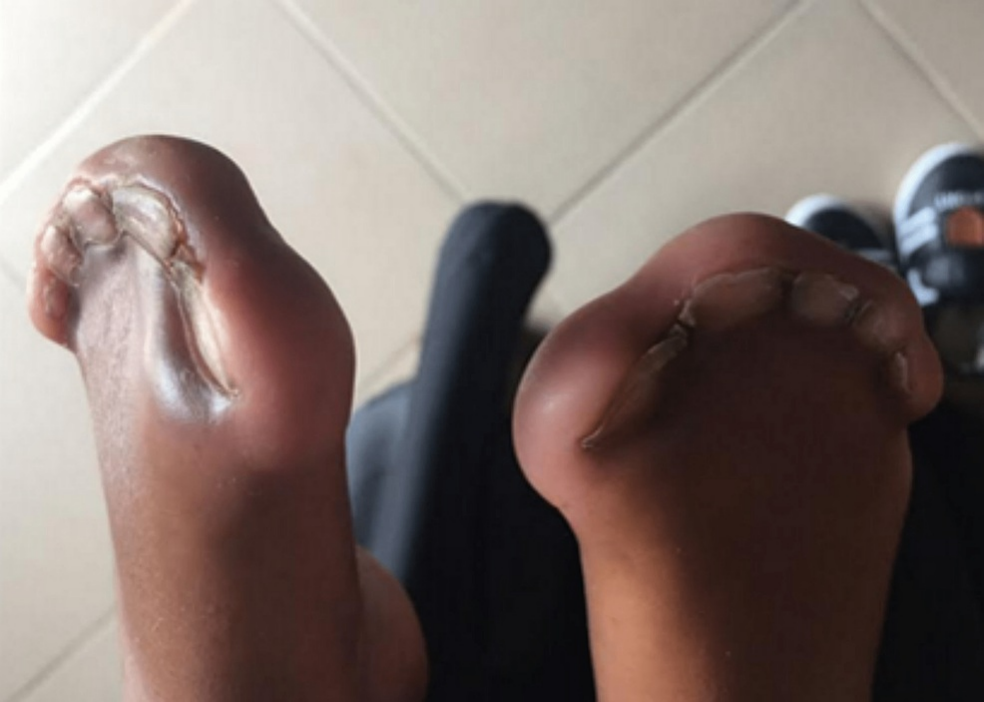
Bilateral symmetrical syndactyly of the feet

**Figure 4:**
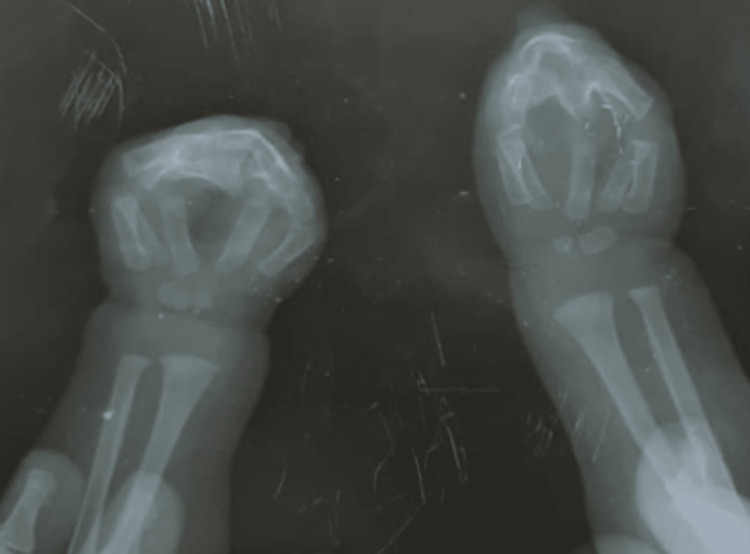
Hand-wrist radiograph showing soft tissue syndactyly and synostosis of the phalanges

The child had type III Apert hands and was fully dependent on his parents for feeding, dressing, and washing. He was unable to manage or hold objects such as spoons, even with two hands. There was no family history of a similar presentation or genetic abnormalities. On examination, he had a prominent forehead, ocular hypertelorism, downward palpebral slant, and a flat nasal bridge with a bulbous nose (Figure 1). His hands and feet both displayed bilateral symmetrical syndactyly with complete fusion of all five digits (Figures 2, 3). Hand-wrist radiography revealed soft tissue syndactyly and synostosis of the phalanges (Figure 4).

**Figure 5:**
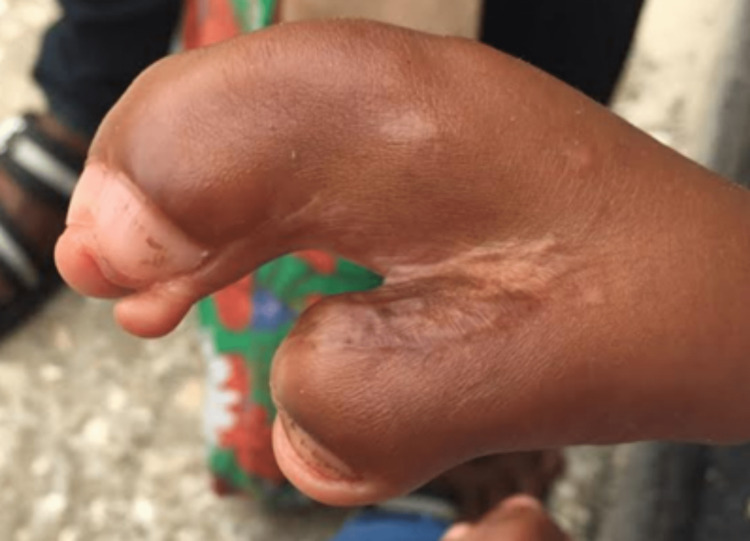
Post stage I (creation of the first web space)

Surgical management involved hand reconstruction in three stages, starting when the boy was three years old. Stage I, conducted in 2019, created the first web space, which would permit grasp (Figure 5). The thumb was carefully separated while protecting the ulnar neurovascular bundle and preserving the ulnar collateral ligament. Opposition of the thumb was achieved followed by centralisation of the tendons. A full-thickness skin graft was taken from the distal forearm via a transverse elliptical incision. Primary closure of the donor site was done. The wound healed with no complications, and after three weeks, a physiotherapy training program was initiated to teach the child how to grasp and hold objects.

**Figure 6:**
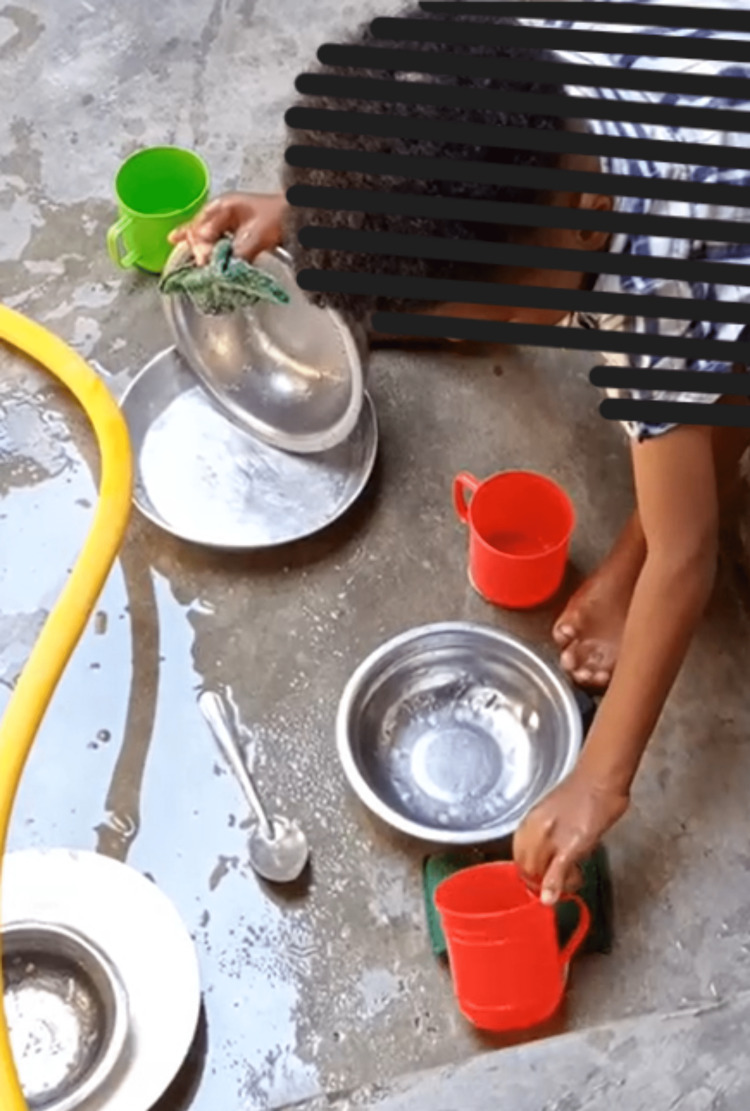
Functional grasp after stage II

Stage II to create the fourth web space commenced in May 2022 after the elevation of COVID-19 restrictions. The same standards as stage I were applied. A skin graft was taken from the same site on the forearm and the skin permitted a primary closure of the donor site. This further improved hand function, enabling the boy to pick up cups and plates (Figure 6).

**Figure 7:**
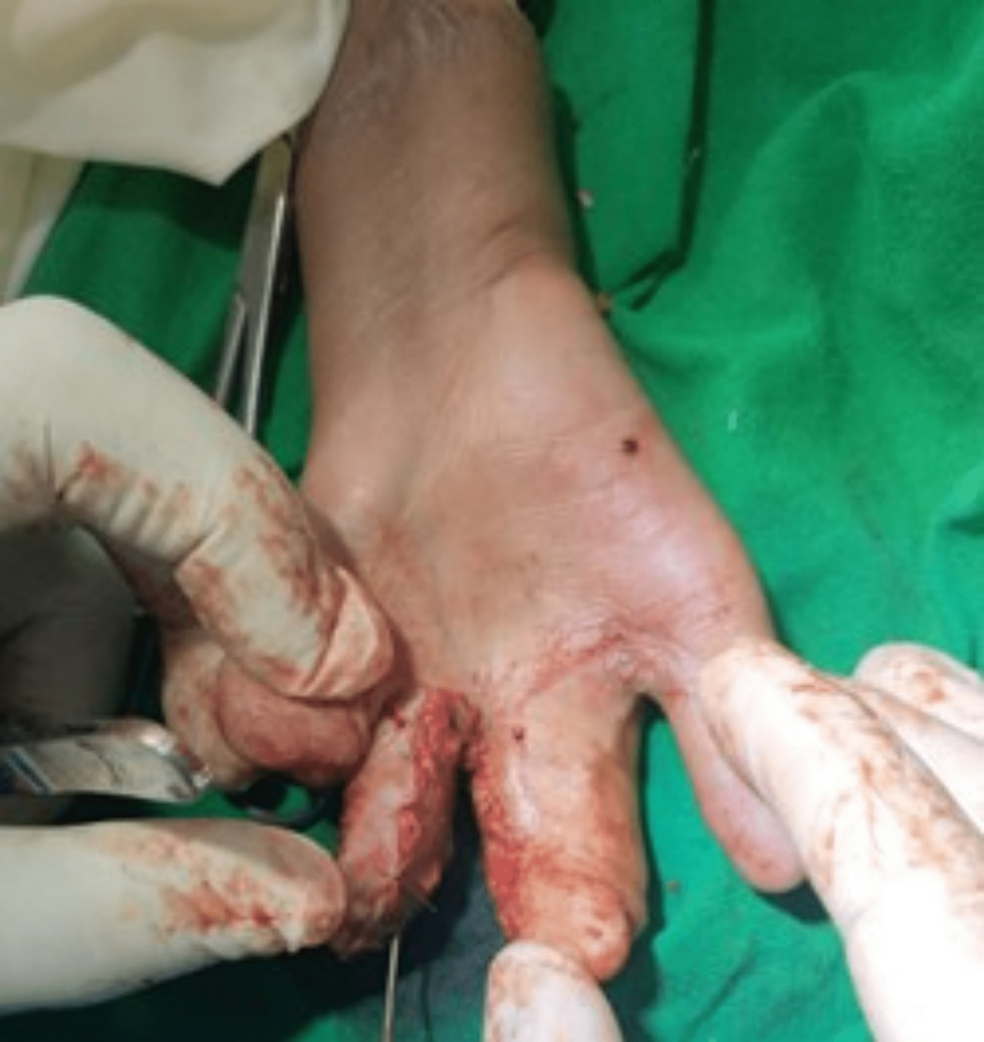
Stage III (creation of the second web space)

**Figure 8:**
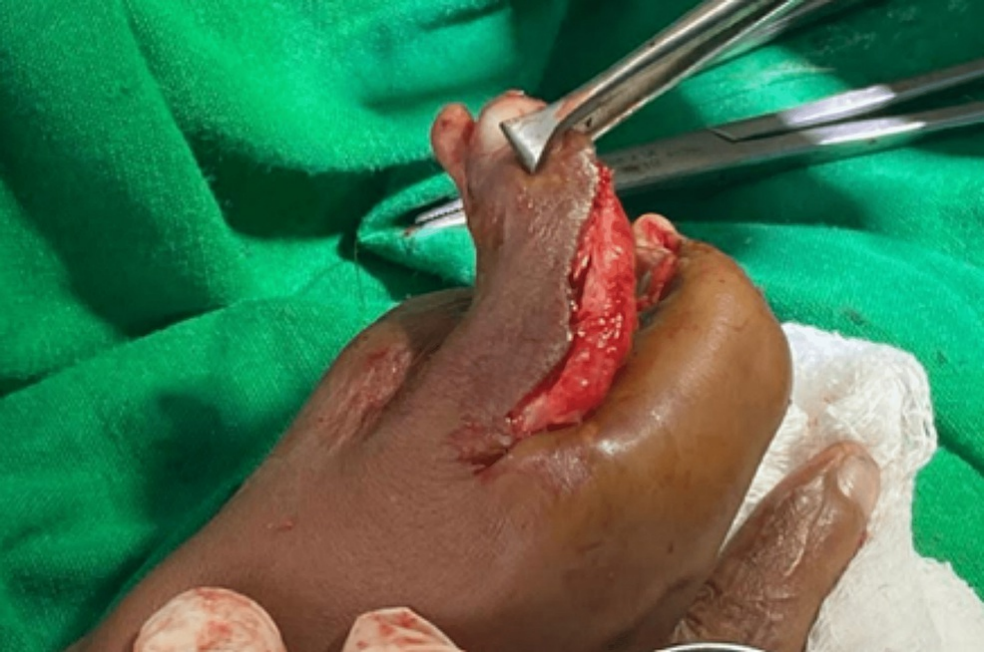
C-shaped index finger with radial convexity

Stage III was carried out in March 2023 to create the second web space (Figure 7). The patient had a C-shaped index finger with radial convexity (Figure 8). This required a radial closing wedge osteotomy through the middle phalanx and an intra-medullary fixation by a 16-gauge cannula because k-wires were unavailable. This was followed by the centralisation of the extensor tendon. The skin graft taken from the right anterolateral thigh was enough for both hands. The donor site underwent primary closure.

**Figure 9:**
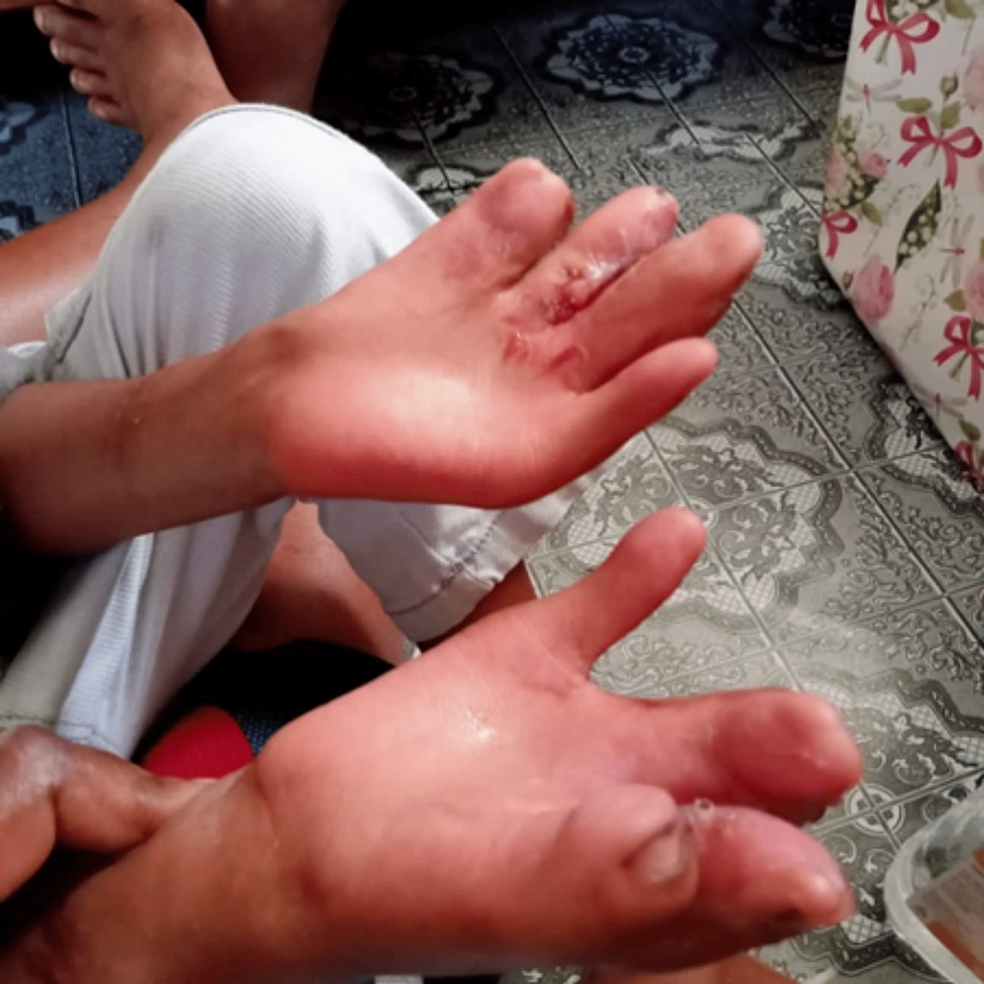
Post-stage III recovery showed uneventful healing

The postoperative period was smooth. After stage III, healing was uneventful (Figure 9). The child cooperated with fine skills hand training and was able to manage with both hands. At four-month post-surgery, his hand grip strength was 60% that of a normal child of the same age. The patient subsequently underwent a hand rehabilitation program. The patient improved with time, and by seven years old, he was able to attend school, hold a pen, and write. The three-stage bilateral hand reconstruction was thus successful in achieving good functional outcomes for the boy.

## Discussion

A literature search was conducted on PubMed using the following combination of keywords: “Apert syndrome” or “Apert hands” and “hand surgery” or “hand reconstruction”. Results were restricted to English publications with available full-texts and case reports involving hand reconstruction surgeries in the management of Apert syndrome without any date restrictions. This search revealed that the literature discussing hand reconstruction in Apert syndrome is scarce compared to the amount of literature on craniofacial surgeries.

Despite the age to initiate surgery ranging from three to 12 months, there is a consensus that the creation of the first web space should be prioritised to facilitate grasp [3,8,9]. In addition to the age range at which hand reconstruction should be initiated, Zucker et al. (1991) further state that digital separation should be achieved by three years old with six-month intervals between operations [8]. Our case describes a boy who started hand reconstruction surgery at three years old while still producing positive functional outcomes. Dao et al. (2002) describe a case of where syndactyly release was initiated when the child was two years old, which yielded good outcomes in hand appearance and function despite the surgery being initiated relatively late [10]. Roje et al. (2012) present seven cases where the children were all above 12 months old, two being above two years old [9]. Despite their ages, all seven children were able to develop a functional pincer grasp, which enabled writing [9]. Carneiro et al. (2008) describe a 19-year-old male with pathognomonic syndactyly of the fingers and toes of Apert syndrome, needing nine surgeries to separate his fingers to permit function of which they were successful, allowing the patient independence in some tasks [2]. Roje et al. (2012) concluded that although the age at which operations commence is an important factor in determining the successful management of complex cases, it is not the main factor for a good outcome [9].

The literature suggests several different techniques for hand reconstruction in the management of syndactyly in Apert syndrome. Zucker et al. (1991) recommend split-thickness skin grafts due to the large surface area of graft needed and that harvesting full-thickness skin grafts may interfere with the vascular supply of any pedicled groin flaps used in the future [8]. Full-thickness skin grafts take longer to harvest and are less successful while split-thickness has a higher incidence of secondary graft contracture [3]. Our case saw success with full-thickness skin grafts. Roje et al. (2012) used inter-digital zig-zag incisions and full-thickness skin grafts taken from the inguinal or buttock areas, dorsal and palmar flaps in quadrangular, triangular or horseshoe shapes to reconstruct the web floor, and additional Z or W-plasty to release concomitant flexion contractures [9]. They also utilised wedge osteotomies in all patients with additional cancellous bone grafts from the iliac crest, as well as a distraction lengthening procedure and bone grafting from the iliac crest or a bone homograft to lengthen short thumbs [9]. Dao et al. (2002) used a radial mid-lateral incision from the level of the distal phalanx to just proximal to the metacarpal-phalangeal joint, which could be converted to a Z-plasty for skin closure [10]. They chose to release the long lever arm of the abductor pollicis tendon instead of using an osteotomy, stating that releasing the abductor pollicis brevis tendon and excising the ulnar metacarpal head prominence makes collateral ligament releases unnecessary and avoiding osteotomies of the proximal phalanx eliminates the risk of injuring existing physis or epiphysis, minimises the risk of injuring the metacarpophalangeal joint, and prevents the radial angulation from recurring [3,10]. Lee et al. (1975) treated a case of typical Apert syndrome using web-plasty of the first and second web spaces of the right hand [11]. Mesang et al. (2019) describe five cases, with ages ranging from 19 months to six years [6]. Surgeries included full-thickness skin grafts, dorsal rectangular flaps, palmar rectangular flaps, V-shaped palmar flaps, zigzag incisions to create interdigital flaps and release web spaces, and Buck-Gramcko pulp flaps to recreate nail folds and osteotomies of fused distal phalanges, without any reported intraoperative complications [6]. Furthermore, Driessen et al. (2017) state that the release and reconstruction of the thumb with desyndactylisation of one or more web spaces during the early stages of reconstructing a five-fingered hand also improves neurocognitive development [1]. The difference in presentations hence requires treatment to be highly individualised [1,2]. To produce desirable functional and aesthetic outcomes, surgical techniques are chosen based on the patient’s condition and the surgeon’s decision [6].

Cases discussed in the literature have generally positive outcomes. Among seven cases analysed by Roje et al. (2012), all had a functional pincer grasp, good aesthetic outcomes, and parent satisfaction, but two struggled with a firm palmar grip because of a symphalangism in the central zone of the hand, requiring arthroplasty with Swanson-type silicon metacarpophalangeal joint prosthesis or an “intrinsic muscle” release with tendon transfer to improve index finger extension [9]. Among two cases discussed by Dao et al. (2002), one case had functional thumbs with minimal radial angulation at the metacarpophalangeal joint, demonstrable tip-to-tip pinch with small fingers and parent satisfaction at 18 months, while the other case had straight thumbs with no lateral angulation at the metacarpophalangeal joint, active tip-to-tip pinch with the small finger on the left but only passive tip-to-tip pinch on the right, good functional and aesthetic outcomes, and parent satisfaction at five years and eight months [10]. Although the exact functional outcomes of patients vary given the differences in their initial presentation and management strategies, most cases have reported thumb function while limitations in hand function are further rectified using innovative hand surgery techniques. Three of the five cases discussed by Mesang et al. (2019) had follow-up outcomes, ranging from five days to four months, all being uneventful [6]. Koca (2016) describes a 19-year-old female presenting with syndactyly consistent with type III Apert syndactyly while also displaying a large thumb consistent with Pfeiffer syndrome and polydactylic toes consistent with Carpenter syndrome, demonstrating the difficulty of differentiating craniosynostosis syndromes [4]. Given that Apert syndrome presents with many symptoms in addition to syndactyly of the hands and feet, it is especially important for a multidisciplinary team of different specialities to collaborate in its management to produce positive outcomes [4]. Social and occupational therapy can also bring great emotional and physical benefits to patients and families [2].

## Conclusions

This case report shows that hand reconstruction surgery in the management of complete syndactyly seen in type III Apert hands is possible and can yield positive results, even when initiated relatively late. Restoration of hand function and achieving developmental milestones can be accomplished and earlier interventions produce better results. Given the rarity of Apert syndrome and the scarcity of literature discussing the reconstruction of Apert hands, there is a divide on the optimum age to initiate surgery and what surgical techniques should be used.

## References

[1] Driessen C, van Veelen MLC, Joosten KFM (2017). Apert syndrome: the Paris and Rotterdam philosophy. Expert Opin Orphan Drugs.

[2] Carneiro GV, Farias JG, Santos FA, Lamberti PL (2008). Apert syndrome: review and report a case. Braz J Otorhinolaryngol.

[3] Pettitt DA, Arshad Z, Mishra A, McArthur P (2017). Apert syndrome: a consensus on the management of Apert hands. J Craniomaxillofac Surg.

[4] Koca TT (2016). Apert syndrome: a case report and review of the literature. North Clin Istanb.

[5] Yacubian-Fernandes A, Palhares A, Giglio A (2005). Apert syndrome: factors involved in the cognitive development. Arq Neuropsiquiatr.

[6] Mesang WI, Budi AS, Hutagalung MR (2019). Surgical technique for complex syndactyly in Apert syndrome: a serial case. J Rekonstruksi Estetik.

[7] Upton J (1991). Classification and pathologie anatomy of limb anomalies. Clin Plast Surg.

[8] Zucker RM, Cleland HJ, Haswell T (1991). Syndactyly correction of the hand in Apert syndrome. Clin Plast Surg.

[9] Roje Z, Roje Z, Ninković M, Dokuzović S, Varvodić J (2012). Reconstruction of the hand in Apert syndrome: two case reports and a literature review of updated strategies for diagnosis and management. Acta Chir Plast.

[10] Dao KD, Shin AY, Kelley S, Wood VE (2002). Thumb radial angulation correction without phalangeal osteotomy in Apert's syndrome. J Hand Surg Am.

[11] Lee HA, Rah SK, Ahn BH (1975). A case of acrocephalosyndactyly. J Korean Orthop Assoc.

